# Expression and bioactivity of human α-fetoprotein in a Bac-to-Bac system

**DOI:** 10.1042/BSR20160161

**Published:** 2017-01-17

**Authors:** Bo Lin, Kun Liu, Wenting Wang, Wei Li, Xu Dong, Yi Chen, Yan Lu, Junli Guo, Mingyue Zhu, Mengsen Li

**Affiliations:** 1 Hainan Provincial Key Laboratory of Carcinogenesis and Intervention, Hainan Medical College, Haikou 571199, Hainan Province, PR China; 2 Key Laboratory of Molecular Biology, Hainan Medical College, Haikou 571199, PR China; 3 Institution of Tumor, Hainan Medical College, Haikou 570102, Hainan Province, PR China; 4 Department of Anesthesiology, Second Affiliated Hospital, Hainan Medical College, Haikou 570311, PR China

**Keywords:** Alpha fetoprotein, Bioactivity, Expressed vectors, Hepatoma cells

## Abstract

α-fetoprotein (AFP) is an early serum growth factor in foetal embryonic development and hepatic oncogenesis. A growing number of investigations of AFP as a tumour-specific biomarker have concluded that AFP is an important target for cancer treatment. AFP also plays an immunomodulatory role in the treatment of several autoimmune diseases, such as rheumatoid arthritis, multiple sclerosis, myasthenia gravis and thyroiditis. In an effort to support biochemical screening and drug design and discovery, we attempted to express and purify human AFP in a Bac-to-Bac system. Two key factors affecting the expression of recombinant human AFP (R-AFP), namely the infectious baculovirus inoculum volume and the culturing time post-infection, were optimized to maximize the yield. We achieved a high yield of approximately 1.5 mg/l of harvested medium with a 72–96 h incubation period after infection and an inoculum volume ratio of 1:100. We also assessed the role of R-AFP in the proliferation of the human liver cancer cell line Bel 7402, and the results indicated that R-AFP promoted the growth of hepatoma cells. We concluded that this method can produce high yields of R-AFP, which can be used for studies related to AFP.

## Introduction

High levels of serum α-fetoprotein (AFP) are associated with embryonic development and cancer growth [1–3]. The *afp* gene is a member of the family of albuminoid genes, including serum albumin (SA), vitamin D-binding protein (VTDB) and α-albumin (afamin). The albuminoid genes evolved from a common ancestor and exhibit considerable similarity in their primary structure. For example, human AFP and human serum albumin (HSA) share 40% identity with highly conserved cysteine residues. Human AFP consists of 609 amino acids, has a molecular mass of 69 kDa and contains only one glycosylation site (N233). However, the glycosylation site may link various carbohydrate moieties, and the structure of the carbohydrate moieties varies in different tissues and diseases [3].

Studies have found that AFP can regulate hepatocellular growth, differentiation, regeneration and transformation in oncogenic growth processes [4–8]. AFP is also an immunomodulatory molecule, as transfer of foetal AFP through the placenta into the mother’s circulation is correlated with remission of rheumatoid arthritis, multiple sclerosis and other autoimmune disorders [9]. Recombinant expression of human AFP is under development as a biopharmaceutical for the treatment of autoimmune diseases [9], and human AFP is also being used as a bioactivated molecule in drug discovery studies for cancer treatment [4–8]. Recombinant expression of human AFP has been described in *Escherichia coli* expression systems [10,11], in yeast [12] and in the milk of transgenic goats [13], but human AFP expression is unsatisfying in these systems. For example, human AFP production in *E. coli* expression systems yields inclusion bodies, and refolding of this material is not practical for commercial production [13]. In addition, human AFP produced in the milk of transgenic goats is not easily purified.

The present study is the first to report a high yield of recombinant human AFP (R-AFP) in a Bac-to-Bac baculovirus expression system. We also detected the bioactivity of R-AFP in the human liver cancer cell line Bel 7402 and found that R-AFP promotes hepatoma cell growth. The present study established a reliable, convenient method for expressing and producing a high yield of R-AFP, which could be used for drug screening and for structural and functional studies.

## Materials and methods

### Expression vector construction

Human AFP (NCBI: NM_001134) with a C-terminal 6× His-tag was cloned into the pFastBac 1 vector (Invitrogen Inc, U.S.A.). After the fusion sequences and the reading frames were confirmed by sequencing, this pFastBac 1 vector construct was transformed into bacterial *DH10* cells, and the extracted bacmid was then transfected into Sf9 cells using Cellfectin II Reagent (Invitrogen Inc, U.S.A.) to obtain passage 1 baculoviruses (P1 baculoviruses) [14].

### R-AFP expression in a Bac-to-Bac baculovirus system

R-AFP was expressed using the Bac-to-Bac Baculovirus Expression System (Invitrogen Inc, U.S.A.). The process was as follows: (1) Sf9 insect cells were cultured in Insect-Xpress protein-free medium (Lonza Group Ltd., Switzerland) without serum at a density of 2 × 10^6^ cells/ml. (2) The P1 baculoviruses were harvested after the transfected Sf9 cells were incubated at 27°C for 7 days. (3) One hundred microlitres of P1 baculovirus were added to 8 ml of Sf9 cells and harvested at 72 h after infection. The baculoviruses were amplified for two rounds to obtain P3 baculoviruses. (4) Ten millilitres of P3 baculoviruses was added to 1 litre of Sf9 cells, and secreted R-AFP was harvested in the medium at 72 h after infection, as described previously [14–18].

### Analysis of the expression of secreted R-AFP

The insect medium (approximately 2 ml) containing secreted R-AFP was collected and centrifuged at 6000 rpm for 15 min. The supernatant was added to 200 μl of 10× HBS buffer (10 mM Hepes [pH 7.2] and 150 mM NaCl) and 80 μl of nickel (Ni)-charged resin (GE Healthcare company, U.S.A.). After the sample was mixed and shaken for 2 h, the 6× His-tag R-AFP in the supernatant was captured by Ni-charged resin and was eluted with 100 μl of 300 mM imidazole in HBS buffer.

The eluted R-AFP was analysed by SDS/PAGE. The reduced protein SDS/PAGE sample contained a reducing buffer, such as DTT, and was boiled for 3 min. The non-reduced protein sample did not contain a reducing buffer and was not heated [18].

### Analysis of cytoplasmic R-AFP

Cytoplasmic R-AFP was analysed as follows: after 72 h incubation, the media were harvested and centrifuged at 3000 rpm for 15 min. Cell pellets were resuspended in 40 ml of HBS buffer, sonicated on ice for 15 min with 3 s/9 s intervals and then centrifuged at 13000 rpm for 60 min. The supernatant was collected, and supernatant containing R-AFP protein was further purified and analysed to determine the expression level of secreted R-AFP as described recently [18].

### Purification of secreted R-AFP in a Bac-to-Bac baculovirus system

After 72 h incubation, the media (approximately 1 litre) containing the secreted R-AFP were harvested and centrifuged at 4000 rpm for 10 min, the supernatant was collected and filtered with a 0.45 µm filter membrane (Millipore Corp., U.S.A). The supernatant was then concentrated to 100 ml by cross-flow filtration (Millipore Corp., U.S.A.), and the buffer was changed to HBS buffer. The concentrate was centrifuged at 10000 rpm for 30 min, and the supernatant was collected and passed through Ni-charged resin (GE Healthcare). R-AFP was captured by the resin and eluted with 300 mM imidazole in HBS buffer. The eluted buffer was concentrated to 1 ml using a 10 kDa filter tube (Millipore Corp., U.S.A.) and then further purified by gel filtration chromatography using a Superdex 200 column (GE Healthcare company, U.S.A.). The detection wavelength was 280 nm. Elution was performed with an HBS buffer at a flow rate of 1 ml/min [18].

### Extraction of human AFP

Human cord blood AFP was precipitated by ammonium sulfate and passed through an anti-AFP affinity chromatography column. AFP-positive fractions were collected and concentrated. The purity of prepared AFP was 92.7% as determined by SDS/PAGE. The protein was stored at −80°C until use [19].

### Western blot analysis of R-AFP

**Separation of protein**: The target protein was electrophoretically separated by SDS/PAGE. **Electrotransfer**: Proteins were transferred from the gel to a PVDF membrane in a constant voltage of 60 V for 2 h at 4°C. **Immunodetection**: Blocking buffer (3%) was added to the PVDF membrane, and the membrane was rocked gently for 2 h. Then, the membrane was rinsed with TBST buffer three times. The appropriate concentration of anti-AFP antibody was added, and the membrane was rocked gently for 12 h at 4°C. The appropriate concentration of horseradish peroxidase-conjugated anti-rabbit antibody was added, and the membrane was rocked gently for 1 h at 37°C. The membrane was then washed with TBST buffer, developing reagent was added, and development was monitored. The antibody against AFP and the horseradish peroxidase-conjugated anti-rabbit antibody were purchased from Sangon Biotech Co., Ltd. (Shanghai) and Jackson ImmunoRes Lab, Inc., (U.S.A.) respectively [5,7,8].

### Optimization of secreted R-AFP expression in a Bac-to-Bac system

The optimization experiments were designed by comparing the effects of two major factors (the infectious baculovirus inoculum volume and the post-infection time) on the production of R-AFP. These experiments were performed by infecting 1-litre cell cultures with baculovirus and incubating the cells at a constant temperature of 27°C under shaking conditions (110 rpm) [14–18]. We harvested the medium after infection at each monitoring time, and the medium was replaced with HBS buffer. The proteins were captured by Ni-charged resin (GE Healthcare Company, U.S.A.) and eluted with 300 mM imidazole in HBS buffer. The eluted 6× His-tagged proteins were concentrated and purified by gel filtration chromatography using a Superdex 200 column. Finally, the proteins were analysed with a NanoDrop 2000 spectrophotometer (at 280 nm) and SDS/PAGE as described recently [14].

### Preparation of a monoclonal antibody against AFP

The monoclonal antibody against human AFP (anti-AFP) was prepared according to standard procedures [20]. Briefly, BALB/C mice were immunized with purified human AFP (Sigma–Aldrich) in complete Freund’s adjuvant at 2- to 3-week intervals. Spleen cells were removed from the immunized mice. Myeloma cells in the exponential phase were mixed with the spleen cells in a certain proportion and fused by 50% PEG3000 buffer. Both cell types were cultured in HAT (H-hypoxanthine, A-aminopterin, T-thymidine) medium supplemented with feeder cells to form a hybridoma cell line. The hybridoma cells that secreted antibody were screened by ELISA and continually cloned by the limited dilution method; thus, a stable anti-AFP antibody producing hybridoma cell line was obtained. BALB/C mice were inoculated with positive clones. Anti-AFP antibody was harvested from ascites fluid and purified by affinity chromatography. The specificity of the monoclonal antibody (with a titre higher than 5000 for AFP) was ascertained by ELISA and Western blot assay to prevent interference from human albumin, which has a structure similar to that of human AFP. The results of the ELISA and Western blot assay showed the specific binding of the monoclonal antibody to human AFP and a lack of reaction to human albumin [19].

### Laser confocal microscopy to observe the expression of human AFP receptor in cells

Expression of the human AFP receptor (AFPR) in the Bel 7402 cell line was examined using a laser confocal microscope as described previously [8]. Briefly, Bel 7402 cells were incubated with a mouse anti-human AFPR antibody (Abcam Biotech Company, Cambridge, U.K.) for 12 h, followed by incubation with secondary goat anti-mouse antibodies conjugated to FITC (Zhongshan Boil Tech Co., Beijing) for 2 h. Then, 10 μl DAPI (1 mg/ml) was added to the mixture. Localization and expression of AFPR in cells were observed and captured by laser confocal microscopy (Leica TCS-NT SP2, Germany).

### Detection of ^3^H-TdR incorporation into cells

Bel 7402 cells were suspended in RPMI-1640 medium containing 10% FBS and were added to a 24-well plate at 1 ml per well followed by incubation at 37°C in a humidified atmosphere of 5% CO_2_ for 48 h. The supernatant was removed and replaced with 150 μl of fresh medium without FBS for another 24 h. Different concentrations of extracted human AFP (E-AFP), R-AFP (0–80 mg/l), HSA or anti-AFP were added to each well for 24 h and then pulsed with 1 mCi of ^3^H-TdR. The cells were harvested on to a glass microfibre filter 4 h later using a multiple sample harvester. The incorporation of ^3^H-TdR was measured using an LKB 1209 Rackbeta liquid scintillation counter. We used E-AFP as a positive control. To determine whether the influence of human AFP on proliferation was specific, the blockage of anti-AFP and HSA as structural analogues was also assessed, and a control group without AFP or HSA was also paralleled to be done, according to the previously described procedure [19].

### Detection of cell growth by MTT assay

A total of 1.5 × 10^4^ Bel 7402 cells per well were plated in 96-well plates and cultured in RPMI-1640 medium supplemented with 10% FBS at 37°C in a humidified atmosphere of 5% CO_2_ for 48 h. The cultured cell medium was replaced with medium without FBS for another 24 h, and the cells were treated with human AFP (20 mg/l) for 48 h. The effects of E-AFP and R-AFP on cell growth were measured by MTT assay as described in a previous study [19]. The growth ratio = (control group *A*_490 _− treated group *A*_490_)/control group *A*_490 _× 100%.

## Results

### Analysis of expression vectors

**Figure 1:**
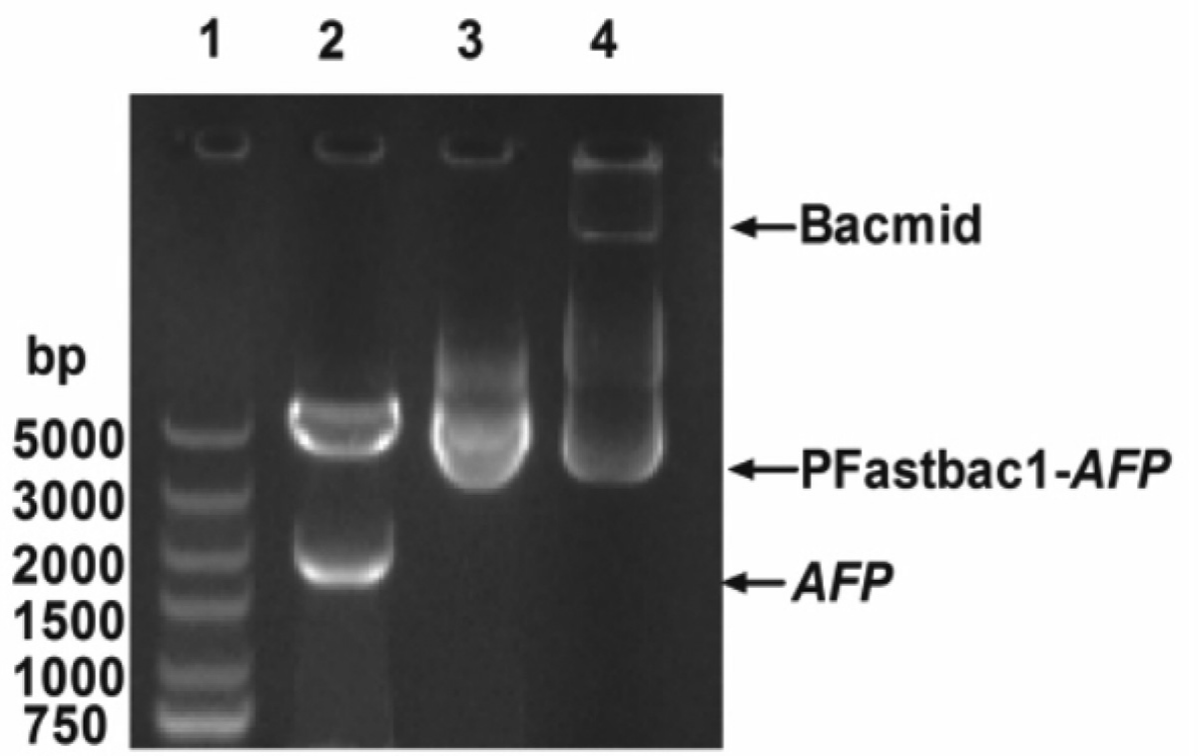
Agarose gel electrophoresis analysis of the recombinant bacmid containing the human *afp* gene Lane 1, DNA marker; lane 2, pFastBac 1–*afp* vector digested by *BamH*I and *Hind*III restriction enzymes; lane 3, pFastBac 1–*afp* vector; lane 4, the recombinant bacmid containing the pFastBac 1–*afp* vector.

The expression vectors were confirmed by agarose gel electrophoresis. Figure 1 (lane 2) shows that the pFastBac 1–*afp* vector could be digested with *BamH*I and *Hind*III restriction enzymes to release a band measuring approximately 2000 bp, confirming the presence of the recombinant human *afp* gene. DNA sequencing of recombinant pFastBac 1–*afp* confirmed the existence of the coding sequence in the correct frame of the vector, without any mutation or alteration in the *afp* gene sequences. Figure 1 (lane 4) shows that the recombinant bacmid contained the pFastBac 1–*afp* vector and confirmed that the correct homologous recombination occurred in the bacmid.

### Analysis of R-AFP baculovirus expression

**Figure 2:**
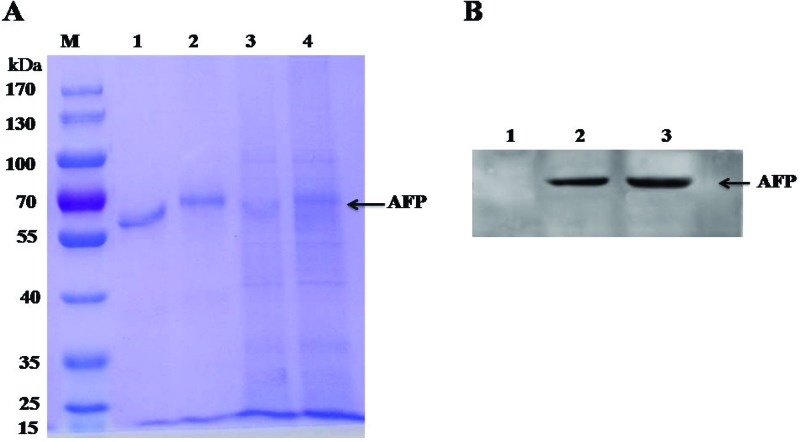
Analysis of baculovirus expression of R-AFP (**A**) SDS/PAGE gel analysis of the baculovirus expression of R-AFP. M, protein marker; lane 1, native secreted R-AFP; lane 2, reduced secreted R-AFP; lane 3, native cytoplasmic R-AFP; lane 4, reduced cytoplasmic R-AFP. (**B**) Western blot analysis of baculovirus expression of R-AFP. HSA and E-AFP are the negative control and positive control respectively. Lane 1, HSA; lane 2, R-AFP; lane 3, E-AFP.

Secreted R-AFP (in the insect medium) and cytoplasmic R-AFP (in the lysed insect cells) were assessed by SDS/PAGE, and the results are shown in Figure 2(A). The non-reduced or native secreted R-AFP band was tilted at approximately 67 kDa, which indicated that the protein was in a non-linear form (lane 1). The reduced protein band (lane 2) was at approximately 69 kDa, consistent with the size of the *afp* gene. The molecular mass of the cytoplasmic R-AFP bands (lanes 3 and 4) was similar to that of the secreted R-AFP bands, but the bands were faint and impure. These results indicated that the cytoplasmic R-AFP concentration was lower than the extracellular R-AFP concentration in the medium and that cytoplasmic R-AFP may bind with other proteins. The R-AFP protein was further evaluated by Western blotting (Figure 2B). The medium containing expressed R-AFP was amplified and used for large-scale expression of the recombinant protein.

### Purification of R-AFP

**Figure 3:**
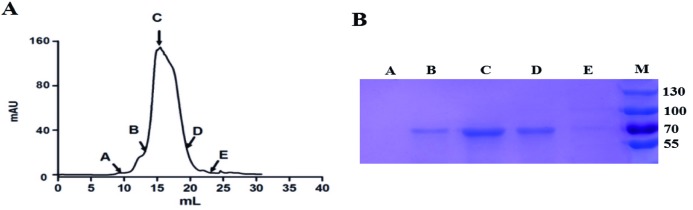
Purification of R-AFP (**A**) Gel filtration chromatography of R-AFP purification. (**B**) SDS/PAGE analysis of the R-AFP purification fractions A, B, C, D and E. The highest peak shows a high concentration of R-AFP (fraction C).

Purification of the secreted R-AFP in the medium is straightforward. First, the medium was concentrated by buffer exchange using HBS buffer. In the second step, R-AFP in the medium was captured on Ni-charged resin, eluted with 300 mM imidazole and purified by gel filtration chromatography. The eluted chromatography position of R-AFP is shown in Figure 3(A) at a elution volume 15.0 ml. The position indicated a molecular mass of 65–70 kDa according to the Superdex 200 column (GE Healthcare) [13]. R-AFP fractions from gel filtration chromatography were collected for further SDS/PAGE analysis. The highest peak showed a high concentration of R-AFP (Figure 3B, lane C), indicating that the purification process was successful. The purified R-AFP is soluble and stable, we test its isoelectric point (IP) is pH 4.5.

### Optimization of R-AFP expression

**Figure 4:**
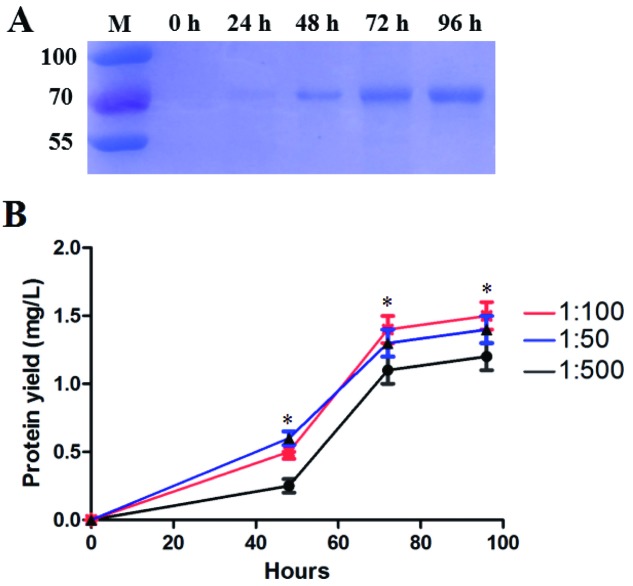
Optimization of the yield of R-AFP (**A**) The R-AFP yields of different expression times from the same batch. (**B**) Optimization of R-AFP expression in different inocula. Sf9 cells were grown in 1-litre shaking flasks to exponential phase (2 × 10^6^ cells/ml). The virus titres were 2 × 10^7^ pfu/ml, with different volumes of the infectious baculovirus inocula (1:50, 1:100 and 1:500) and were monitored at different times (0, 24, 48, 72 and 96 h). R-AFP protein expression reached the highest level of approximately 1.5 mg/l at a 1:100 volume of the infectious baculovirus inocula when harvested at 96 h post-infection. *n*=10, *P<0.05 compared with different infectious baculovirus inoculas 1:500 volume groups.

In the process of analysing R-AFP expression, it was found that different conditions led to different expression levels. Figure 4(A) shows the R-AFP expression yield in samples at different times. After 72 h, the expression yield increased slowly. To optimize the yield of R-AFP protein, the parameters were evaluated systematically under the same virus titres (2 × 10^7^ pfu/ml) and the same Sf9 cell density (2 × 10^6^ cells/ml). The infectious baculovirus inoculum volumes (1:50, 1:100 and 1:500) and R-AFP production were monitored at different post-infection times (48, 72 and 96 h). R-AFP protein expression reached the highest level (approximately 1.5 mg/l of medium) at a 1:100 volume of the infectious baculovirus inocula when harvested at 96 h post-infection, the expression of R-AFP in 1:100 volume of the infectious baculovirus inocula has statistical difference compared with 1:500 volume groups (*P*<0.05) (Figure 4B, red line).

### The bioactivity of R-AFP

**Figure 5:**
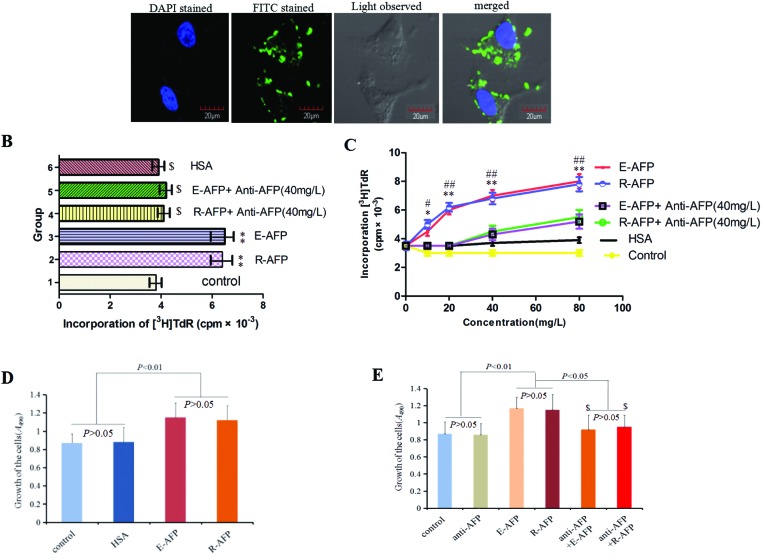
The effects of R-AFP, E-AFP, anti-AFP and HSA on the incorporation of ^3^H-TdR into DNA and proliferation of Bel 7402 cells (**A**) The expression of human AFPR in Bel 7402 cells, which were treated with R-AFP (20 mg/l) for 24 h and observed by laser confocal microscopy. The presented images are from three independent tests. (**B**) The effects of R-AFP (20 mg/l), E-AFP (20 mg/l), anti-AFP (40 mg/l) and HSA (20 mg/l) on the incorporation of ^3^H-TdR into DNA in Bel 7402 cells. 1. Control, 2. R-AFP (20 mg/l), 3. E-AFP (20 mg/l), 4. R-AFP (20 mg/l) + anti-AFP (40 mg/l), 5. E-AFP (20 mg/l) + anti-AFP (40 mg/l), 6. HSA (20 mg/l). ^$^*P*>0.05 compared with control groups; **P<0.01 compared with control groups, HSA groups, R-AFP + anti-AFPgroups, E-AFP + anti-AFP groups. (**C**) The effects of different concentrations (10–80 mg/l) of R-AFP, E-AFP and HSA on the incorporation of ^3^H-TdR into DNA and proliferation of Bel 7402 cells. *P<0.05 and **P<0.05 compared with control groups, ^#^*P*<0.05 and ^##^*P*<0.01 compared with HSA groups, R-AFP + anti-AFPgroups, E-AFP + anti-AFP groups. (**D**) The effects of R-AFP (20 mg/l) and E-AFP (20 mg/l) on the growth of Bel 7402 cells were analysed by the MTT method. (**E**) The blockage effects of anti-AFP to R-AFP and E-AFP on the proliferation of Bel 4702 cells. ^$^*P*>0.05 compared with control groups and anti-AFP groups; *n*=6.

To detect the bioactivity of R-AFP, in the present study, we selected the human liver cell line Bel 7402 to examine the effects of R-AFP on cellular proliferation. First, we observed the expression of AFPR, and the results revealed that Bel 7402 expressed AFPR and that the receptor was located in the cell membrane (Figure 5A). Second, Bel 7402 cells were treated with R-AFP or E-AFP (20 mg/l) for 24 h, and the ^3^H-TdR incorporation results indicated that after treatment with R-AFP or E-AFP, the content of ^3^H-TdR in Bel 7402 cells was markedly enhanced (Figure 5B, groups 2 and 3 respectively). We also selected R-AFP or E-AFP (20 mg/l) co-treated with a human AFP antibody (anti-AFP). The results showed that anti-AFP (40 mg/l) antagonized the effects of R-AFP or E-AFP (Figure 5C, groups 4 and 5 respectively). When we changed the concentration of R-AFP or E-AFP (10–80 mg/l), the results indicated that the ^3^H-TdR content in the Bel 7402 cells was markedly enhanced in a dose-dependent manner. The peak of the increase reached 123.0% compared with the control group (Figure 5C, the red and blue lines). Anti-AFP (40 mg/l) antagonized the effects of R-AFP and E-AFP, but as the concentration of R-AFP or E-AFP increased, the antagonizing effect decreased (Figure 5C, green and purple lines). Further, we used the MTT method to analyse the effects of R-AFP or E-AFP (20 mg/l) on the proliferation of Bel 7402 cells, and the results also demonstrated that R-AFP or E-AFP stimulated the proliferation of Bel 7402 cells (Figure 5D). In addition, anti-AFP reversed the effects of R-AFP or E-AFP (Figure 5E). These results demonstrated that the effects of R-AFP on cellular proliferation are similar to those of E-AFP. HSA did not show an obvious influence on the proliferation of Bel 7402 cells.

## Discussion

R-AFP expressed in the Bac-to-Bac system is soluble and stable as a monomer, and its bioactivity is similar to human AFP extracted from blood. Its IP is pH 4.5. To extract human AFP, a great deal of human cord blood is needed, which is difficult to purify. Expressing R-AFP in the Bac-to-Bac system allows a large amount of purified bioactive protein to be obtained without the need for human cord blood. R-AFP may allow the study of the biochemical features of human AFP and drug screening. Human AFP expression was previously reported in *E. coli* expression systems, in yeast and in the milk of transgenic goats, but these expression systems produced low amounts of human AFP. For example, human AFP expressed in *E. coli* was deposited in bacterial inclusion bodies and subjected to harsh denaturants [10,11,21]. Human AFP expressed in yeast was indistinguishable immunologically from authentic human AFP, and the human AFP produced in yeast contains seven extra amino acid residues at the N-terminus that are not present in mature human AFP [12]. The purification of human AFP expressed in the milk of transgenic goats was difficult and time consuming [13]. There is no such problem when human AFP produced in the Bac-to-Bac system. The Bac-to-Bac system had achieved successful protein expression levels for potential drug applications [16]. The R-AFP produced in the Bac-to-Bac system is highly expressed and easily purified, and its bioactivity is similar to that of the native human AFP. This finding will aid clinical research on the immunomodulatory function of this protein.

To optimize the yield of the R-AFP protein, the post-infection time was considered. Recombinant protein was expressed intracellularly and needed time to be modified and transported to the extracellular space. Thus, the best harvest time depends on the protein and the experiment [14]. In the optimization of R-AFP expression, we found that many cells died and were lysed after 96 h. Therefore, the best harvest time was approximately 72–96 h.

The effects of extracellular human AFP on cellular proliferation are mainly achieved through signal transduction mediated by AFPR. Previously, we found that Bel 7402 cells express AFPR [8]. In the present study, we found that AFPR was highly expressed in the membrane of Bel 7402 cells. This result provided further evidence that the effects of extracellular human AFP on the proliferation of Bel 7402 cells were mediated by membrane-bound AFPR. Previously, we found that human AFP bound to AFPR might activate cAMP and Ca^2+^ to stimulate the transduction of cellular signals to stimulate N-ras and c-myc expression, leading to promote proliferation of NIH 3T3 cells and Bel 7402 cells [19,22]. In the present study, R-AFP and E-AFP played similar roles in the proliferation of Bel 7402 cells, suggesting that R-AFP harbours biological characteristics identical with those of E-AFP. Human AFP is a protein that is specifically expressed in human hepatocellular carcinoma (HCC) cells. We previously found that cytoplasmic human AFP could interact with signal molecules, such as caspase-3 [7], retinoic acid receptor-β (RAR-β) [23] and phosphatase and tensin homologue (PTEN) [5]. These interactions inhibited the transduction of apoptotic signals and activated growth signals, which led to the promotion of proliferation or drug resistance in liver cancer cells. These results suggested that human AFP may interact with these signalling molecules. In the present study, we successfully expressed and purified human AFP protein. We also found that the bioactivity of human AFP protein is similar to that of cytoplasmic human AFP. Recombinantly expressed human AFP is soluble, is stable and can be used to identify signalling molecules or potential drug interactions by surface plasmon resonance (SPR) [24,25]. In conclusion, the present study demonstrated the successful expression and purification of R-AFP, which paves the way for future structural and functional studies of human AFP.

## Abbreviations

AFP: α-fetoprotein
AFPR: human AFP receptor
E-AFP: extracted human AFP
HCC: hepatocellular carcinoma
HSA: human serum albumin
IP: isoelectric point
PTEN: phosphatase and tensin homologue
R-AFP: recombinant human AFP
RAR-β: retinoic acid receptor-β
SA: serum albumin
SPR: surface plasmon resonance
VTDB: vitamin D-binding protein
HBS: HEPES(4-(2-hydroxyethyl)-1-piperazineethanesulfonic acid)
TBST: Tris Buffered Saline with Tween-20
RPMI: Roswell Park Memorial Institute
